# Transcriptome Analysis Reveals the Effects of Troxerutin and Cerebroprotein Hydrolysate Injection on Injured Spinal Cords in Rats

**DOI:** 10.1155/2020/3561235

**Published:** 2020-08-04

**Authors:** Linbang Wang, Hucheng Wang, Ke Tang, Weiyang Zhong, Zhiyu Chen, Zhengxue Quan

**Affiliations:** Department of Orthopedic Surgery, The First Affiliated Hospital of Chongqing Medical University, Chongqing 400016, China

## Abstract

Spinal cord injury (SCI) is a serious condition that results in disability and has a high morbidity rate; its treatment is very difficult. Although troxerutin and cerebroprotein hydrolysate (TCH) injections have been extensively used in clinics in China for the treatment of traumatic brain injury (TBI) and cerebral stroke, the potential efficacy of TCH injection in the treatment of SCI has never been revealed. In this study, the effects of administering TCH injections on neurological recovery in post-SCI rats were first tested with regard to the behavior and histology; subsequently, the specific expression profile of mRNAs and long noncoding RNAs (LncRNAs) in their spinal cords were conducted using RNA sequencing (RNA-seq). The LncRNA-mRNA networks were also elucidated. After SCI, we found that TCH injection with the right dose is effective for the recovery of locomotion function and repairing of the damaged tissue in the spinal cord; TCH injection is also discovered to have a role in the regulation of 443 differentially expressed genes (DEGs) and 27 differentially expressed LncRNAs (DELs) that are identified to have multiple functions, including locomotion, blood vessel morphogenesis, thiamine metabolism, Hippo signaling pathway, and axon guidance, by applying the Kyoto Encyclopedia of Genes and Genomes (KEGG) pathway analysis and Gene Ontology (GO) analysis. In addition, it is revealed that, after SCI, the highly expressed LncRNA AABR07071383.1 in the post-SCI cis/trans-regulates the expression of mRNA Acpp mRNA that encodes a key enzyme involved in the metabolic process of thiamine in the abirritation of the dorsal root ganglion (DRG), which implies that TCH injection may be more effective when administered with benfotiamine (a common treatment drug).

## 1. Introduction

Spinal cord injury (SCI) mostly occurs due to a traumatic event that causes severe damage to the central nervous system (CNS). It results in various degrees of motor and sensory dysfunction [1, 2], high mortality, and multiple complications such as neuropathic pain, syringomyelia, and spasticity, thereby inflicting suffering to more than 40 thousand people worldwide each year [3, 4]. The treatment of SCI has been a challenging topic for the last 100 years, and people are still working towards revealing its mechanism while also making incremental contributions toward function recovery after SCI [5–7].

Troxerutin, also known as vitamin P4, has been proven to possess antihyperlipidemic, anti-inflammatory, antioxidant, and neuroprotective properties [8]. Cerebroprotein hydrolysate, an affluent of peptides and amino acids [9], has been found to have a synergistic effect, along with troxerutin, due to its ability to pass through the blood-brain barrier easily. It has also been known to regulate the metabolism and development functions of neurons to protect against acute ischemic stroke and traumatic brain injury (TBI) [10, 11]. Troxerutin and cerebroprotein hydrolysate (TCH) injection contains 40 mg/ml troxerutin and cerebroprotein hydrolysate with a total nitrogen content of 0.5 mg/ml has been approved and is widely used in the clinical treatment of TBI [12], although the pharmacology of the effects of TCH on traumatic CNS injury at the transcriptome level is still unclear, and there is a lack of direct evidence showing that TCH injection has a positive effect on the recovery of the spinal cord injury.

**Figure 1:**
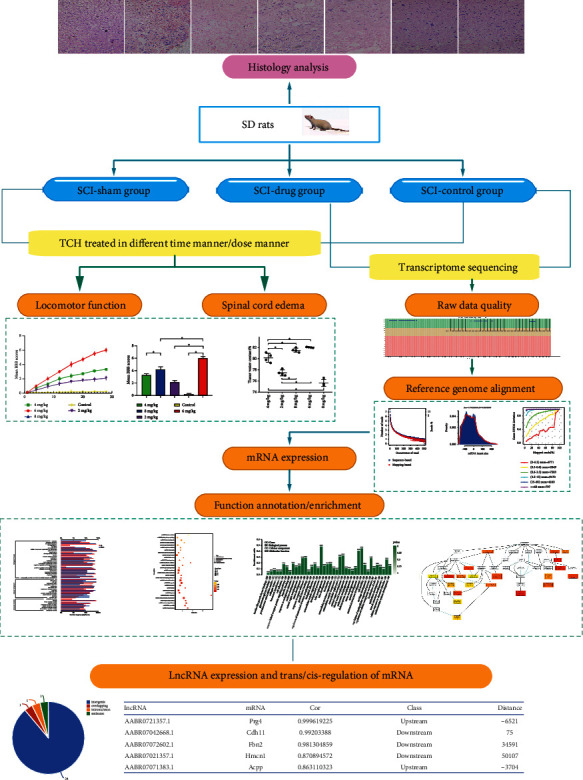
A flowchart of the systematic profile of the effect and transcriptome regulation of TCH application in post-SCI SD rats in the present study.

Therefore, we first applied hematoxylin and eosin (H&E) staining to the damaged section of the spinal cord in a cross manner to verify the angiogenesis and neural restoration effect of TCH injection. Subsequently, we tested the recovery of the motor function of rats by applying the Basso, Beattie, and Bresnahan (BBB) scale [13]. RNA-seq provides the transcriptomic characterization of an individual's activity in a spatiotemporal-specific manner; thus, we did a transcriptome analysis on the rats that were treated with TCH injection after SCI. We found that over 9 genes and LncRNAs were highly involved in the recovery process of SCI. We performed GO and KEGG analyses along with other bioinformatic analyses of the differentially expressed genes and found that TCH is highly concerned with molecular biological functions including the metabolism of thiamine, regeneration of axon, and process of pain. The high expression of LncRNA AABR07071383.1 in the TCH treated group indicates that benfotiamine could be a potential drug that plays a synergistic effect with TCH by trans/cis-regulation of LncRNA [14]; this indicates that a potential drug target is a strategy for clinical treatment. A flowchart of the systematic profiling of this study design and the processed results is shown in Figure 1.

## 2. Materials and Methods

### 2.1. Animal

Adult specific-pathogen-free female Sprague Dawley rats (8 weeks old) were purchased from Laboratory Animal Center of Chongqing Medical University (certificate: SCXK(YU)2018-0003).

The rats were maintained under optimal environment: normal day/night lighting schedule, the relative humidity of 60 ± 10%, a temperature of 25 ± 1°C, and free access to food and water according to the institutional guidelines for the care and use of laboratory animals.

All procedures on the animals of this study were approved by the Ethics Committee Chongqing Medical University (approval no: 2017-128).

### 2.2. Surgery and Drug

The Sprague Dawley rats were divided into the following 6 groups (*n* = 20 per group): SCI control group (control, represented as SCI-CONs in the transcriptome analysis), sham operation control group (Sham), and 4 other experimental groups that were grouped based on their TCH dose (2 ml/kg, 4 ml/kg, 6 ml/kg, and 8 ml/kg). The group that was administered a dose of 6 ml/kg was shown to have the best effect based on the results of function recovery and tissue repairment and was later chosen for the experimental group for RNA sequencing (SCI-DRUGs). The Sprague Dawley rats were preoperatively shaved and anesthetized by an intraperitoneal injection of 1% pentobarbital. Laminectomy is subsequently performed to expose the T10 level of the dura of the spinal cords. In the control and experimental groups, Allen's impactor (provided by the Orthopedics Center, First Affiliated Hospital, Chongqing Medical University) was set at 15 g × 20 cm; one impact was executed in each operation and the success of modeling was judged by both mass morphology of the edema and hemorrhage of the local spinal cord tissue, as well as the spasmodic swing of tails and flutter of hind limbs. The muscle and skin were subsequently sutured by layers. During postoperative care, the rats were permitted to have free access to food and water, and the bedding in cages was changed every day. All rats were intramuscularly injected with penicillin (50,000 IU/times) each day and manual auxiliary urination was performed 3 times a day for the first 3 days.

The rats in the experimental groups were given TCH (batch number. 160602; Buchang Pharmaceutical Co., Ltd., Heze, China), 1 time per day by tail intravenous injection with 0.9% sodium chloride. The rats in the control and sham group were given an equal volume of 0.9% sodium chloride under an identical administration regimen.

### 2.3. BBB Scale

The BBB scale of rats was evaluated before operation, at 3 hours, and every 4 days afterward until 4 weeks after successful modeling to assess the locomotion function recovery of the hind limbs [13]. The scores on the BBB scale increase as the ability to perform fine hind limb movements improves.

### 2.4. Measurement of Spinal Cord Edema

The water content of the injured spinal cord segment tissue was measured by a wet-dry method for the degree of spinal cord edema. Four rats from each group were anesthetized and sacrificed by cervical dislocation on the third day after injury. Spinal cord tissue of about 3 segments (1 cm in length) centered on the lesion was carefully removed. The samples were weighed as wet weight (WW), oven-dried to a constant weight, and weighed as dry weight (DW). The percentage of water content was calculated according to the following formula: content (%) = (1 − DW/WW) × 100% [15].

### 2.5. H&E Staining

Six rats from each group were anesthetized at 12 hours and 2 weeks after injury. The heart was exposed by opening the chest, and 0.9% sodium chloride containing heparin (15 U/mL) was injected into the left ventricle until the body stiffened, followed by perfusion with 4% paraformaldehyde (Servicebio Co., Ltd., Wuhan, China). Spinal cord tissue of about 3 segments centered on the lesion was carefully removed and placed in 4% paraformaldehyde for 36 hours. The paraffin embedding was subsequently operated on and cut into 6 slices of 6 *μ*m thickness by microtome (Servicebio Co., Ltd., Wuhan, China). The slices were heated (60°C for 30 minutes) to melt the paraffin wax, deparaffinized in xylene (Servicebio Co., Ltd., Wuhan, China), dehydrated in ethanol, and stained by H&E (Servicebio Co., Ltd., Wuhan, China). These slices were subsequently differentiated with 1% hydrochloric acid, stained with eosin (Weigesi Biotechnology Co., Ltd., Guangzhou, China), cleared in xylene, mounted with neutral resin, and finally observed using an optical microscope.

### 2.6. RNA Isolation and Library Construction

Two rats from each group were anesthetized and sacrificed by cervical dislocation on the 14th day after injury. Spinal cord tissue of about 3 segments centered on the lesion was carefully removed, and the total RNA isolation process was conducted immediately by using an RNA Extraction Kit (Takara RNAiso Co., Ltd., Beijing, China). RNA concentration was confirmed to be higher than 200 ng/*μ*L, and the quality was tested with Nanodrop2000 (Thermo Fisher Scientific, Inc., Waltham, MA, USA); OD260/280 was between 1.8 and 2.2. The criterion for the qualification of RNA-seq was the RNA integrity number (RIN value), which was verified using Agilent2100.

Illumina TruseqTM RNA Sample Prep Kit I (Illumina, Inc., San Diego, CA, USA) was used for library construction [16]; to be noted, the rRNA in the system was firstly removed by using RNA Purification Reagent (EpiCentre, Inc., San Diego, CA, USA) as a portion of the LncRNA in the cell (>24%) lacks poly A tails so that more detailed and complete results of the LncRNAs expression can be obtained, UNG enzyme (Illumina, Inc., San Diego, CA, USA) was used for cDNA synthesis, TBS380 Picogreen (Invitrogen; Thermo Fisher Scientific, Inc., Waltham, MA, USA) was used for quantification of cDNA, Certified Low Range Ultra Agarose (Bio-Rad Laboratories, Inc., Hercules, CA, USA) was used for the library collection, cBot Truseq PE Cluster Kit v3-cBot-HS (Illumina, Inc., San Diego, CA, USA) was used for bridge amplification, and Hiseq2000 Truseq SBS Kit v3-HS (200 cycles, Illumina, Inc., San Diego, CA, USA) was used for sequencing.

### 2.7. Process of Reads and Identification of Differentially Expressed Genes

The quality of raw data is evaluated in 3 steps: first, the base mass distribution statistics is obtained using FastQC (version v0.11.4), then the raw data are cut and filtered using Cutadapt (version v1.16, parameter is set to -q 20 -m 20), and finally, alignment analysis of sequence data to reference genome is performed using HIST2 (version v 2.1.0, with parameters set to -p 10 and rna-strandness RF). The quality assessment of the sequencing library was performed using the read repeatability assessment, expression saturation evaluation, and insert fragment length check using RSeQC (version v2.6.4). StringTie (version v1.3.3b) was used to assess the expression level, and edgeR (Version v 3.24) was used for DEGs filtering. DEGs clustering, GO/KEGG annotation, and GO/KEGG enrichment analysis were done using software plot_cluster_exp (version is v 1.1.0), GO_anot_exp (version is v 1.4.0), and GO/KEGG_enrichment (version is v 2.1.0), respectively. The method of analyzing the expression and function of LncRNA is the same as that of the DEGs, cis-regulation of LncRNAs-mRNAs was done using LncRNA_mRNA_targets (version v 2.1.0), and trans-regulation of LncRNAs-mRNAs was done using RNAplex (version v2.1.8).

### 2.8. Statistical Analysis

SPSS 19.0 statistical software (IBM, Armonk, NY, USA) was used to analyze the data. All data were expressed as mean ± standard deviation. The differences between the groups were analyzed by one-way analysis of variance or Student's *t*-test. *p* value < 0.05 was considered a statistically significant difference.

## 3. Results

### 3.1. Assessment of Locomotion Function in Post-SCI Rats with Different Treatments

**Figure 2:**
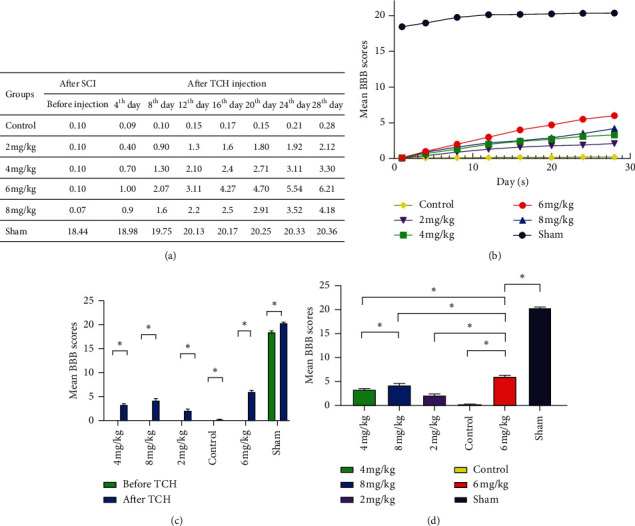
Behavioral performance of the rat model of spinal cord ischemia-reperfusion injury at different time points following surgery. (a) BBB scale scores. (b) Dynamic evaluation of the scores in each group, *p* < 0.05. (c) Experimental groups compared to the control groups on the twenty-eighth day, *p* < 0.05. (d) The 6 ml/kg group compared to other experimental groups and sham group, *p* < 0.05.

The results of the BBB scale in all groups before and after injury are illustrated in Figure 2. The motor function of the rats in the sham group was largely unaffected, whereas motor function in other groups was completely lost after modeling (Figure 2(b)). BBB scores at 1, 4, 8, 12, 16, 20, 24, and 28 days in all experimental groups were higher than the scores prior to administering a TCH injection (Figures 2(b) and 2(c)), and the 6 ml/kg group had higher scores than other experimental groups at 8, 12, 16, 20, 24, and 28 days (*p* < 0.05; Figures 2(b) and 2(d)).

**Figure 3:**
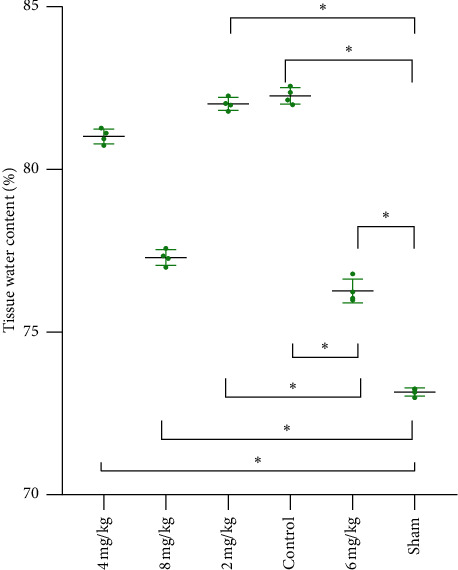
Spinal cord water content at 72 hours after SCI (*n* = 4). Each point in the graph shows the percentage of water per spinal cord (*n* = 4 per group).

The water content of spinal cords was measured on the third day after the injury as edema of the spinal cord developed to a peak after SCI. As shown in Figure 3, each group, when compared with the sham group, showed obvious edema (*p* < 0.05), and tissue water content of the rest groups reduced poorly (*p* < 0.05) compared to the 6 ml/kg group except for the 8 ml/kg group (*p* > 0.05).

**Figure 4:**
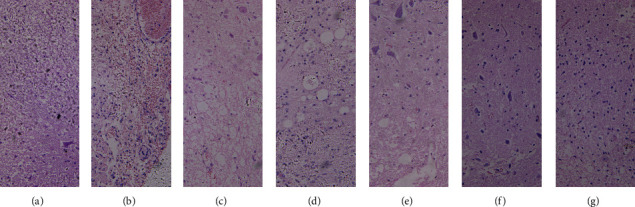
Pathological H&E staining of spinal cord tissue in each group (×100). (a)–(g) The sham group, experiment group (6 hours after operation), control group, 2 ml/kg group, 4 ml/kg group, 6 ml/kg group, and 8 ml/kg group, respectively. (a) Neurons were smooth and arranged well without absence. (b) Obvious hemorrhage. (c) Plenty of space among neurons was clearly visible with severe necrosis. (d) Neurons were reduced obviously. (e) Neurons were arranged densely. (f) and (g) Neurons arranged well.

The results of H&E staining showed that the neurons of the sham group were smooth and well arranged, without absence and morphological changes; the cells in the SCI and control groups both reduced measurably with severe necrosis and swelling; both the 6 ml group cells and the 8 ml group cells arranged well, and the former was without obvious edema (Figure 4).

### 3.2. Transcriptome Sequencing and Data Quality Control

Raw data obtained from Illumina sequencing were primarily transformed into FASTQ format, in which the base and its mass fraction of reads could be recorded. The results of each sample are shown in Table 1; it was observed that the error rate of the base was below the upper limit for subsequent analyses, the content of GC-content percentage was normal, and the data size was permissible for LncRNAs recognition and analysis. The quality of reads was assessed by base mass distribution analysis and represented by quality value (*Q*) as shown in Figure S1; *Q* declined as the process of sequencing progressed since the enzyme activity and sensitivity of the reactants reduced; however, all our samples were qualified for subsequent analyses.

The original sequencing data were filtered since the raw sequencing data contained sequencing linker sequences, low-quality reads, high-N sequences, and short-length sequences that could seriously affect the quality of subsequent assembly. The data statistics before and after (represented as clean, C; and raw, R) the quality trimming is shown in Table 1.

### 3.3. Comparison Analysis with Reference Genome and Quality Control of Transcriptome Library

The transcriptome sequencing data were aligned to the data of the reference genome for subsequent analyses. The reads that could be aligned were named mapped as reads, and the performance of the genome assembling could be illustrated by the proportion of mapped reads in all clean reads. This was named as alignment efficiency, and the alignment result is shown in Table 2. The alignment efficiency was above 70% and was in accordance with the requirements for subsequent analyses.

**Figure 5:**
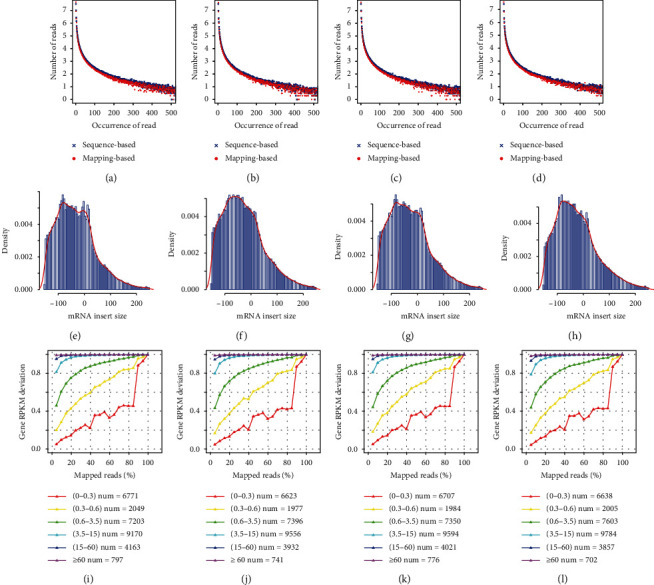
Assessment of sequencing library quality. As the number of repetitions increases, the number of log 10 sequences corresponding to the number of repetitions decreases, and the overall trend of each group is extended linearly and smoothly as shown in (a)–(d). The density of different distances between the double-ended reads on the reference genome ranges from -150 bp to 250 bp, and the main peak of each sample is near 0 bp in (e)–(h). The peak shape is wide, indicating that the length of the insert is more discrete. The gene expression saturation curve of different expression levels is illustrated with a different color for each line in (i)–(l): red lines represent 0–0.3, yellow lines represent 0.3–0.6, green lines represent 0.6–3.5, light blue lines represent 3.5–15, and purple lines represent ≥60. The percentage of effective alignment reads increases along with the proportion of the expression with an error rate of under 15% compared to the final value: the closer the proportion value is to 1, the more saturated the expression level tends to be. The sooner the plateau is reached, the higher is the expression level of the gene in the sample.

Two strategies were applied to evaluate the repetitive rate: one was based on the sequence such that the exact same reads that recurred were recognized as repetitive reads, and the other was based on the alignment such that the reads that were aligned on the same starting site of the reference genome and had the same splicing pattern were considered as repetitive reads, as shown in Figures 5(a)–5(d). Subsequently, the inserting segment length was verified, and a cross-intron segment with a greater length was aligned to the reference genome since the mature mRNA in eukaryons did not contain introns. The inserting segment length was counted as the distance between the start and endpoints of the reads inserted on both sides of the segments in the reference genome. The dispersion degree was used to evaluate the results of the magnetic beads purification procedure as shown in Figures 5(e)–5(h). Then, the expression saturation assessment was performed; the transcripts of genes at different expression levels required different amounts of data volume to be effectively detected. For example, a relatively high-expression gene would require less data volume to approach saturation point, while low-expression ones require larger data volumes to ensure detection accuracy as observed in Figures 5(i)–5(l), the sample had a high degree of sequencing saturation, and most of the genes with medium or above expression (i.e., genes with FPKM values above 3.5) were close to saturation at 20% alignment of the sequencing reads (the vertical axis value approached 1). This indicated that the overall quality of saturation was high and that the amount of sequencing covered most of the expressed genes.

### 3.4. Differential Expression Gene Analysis

**Figure 6:**
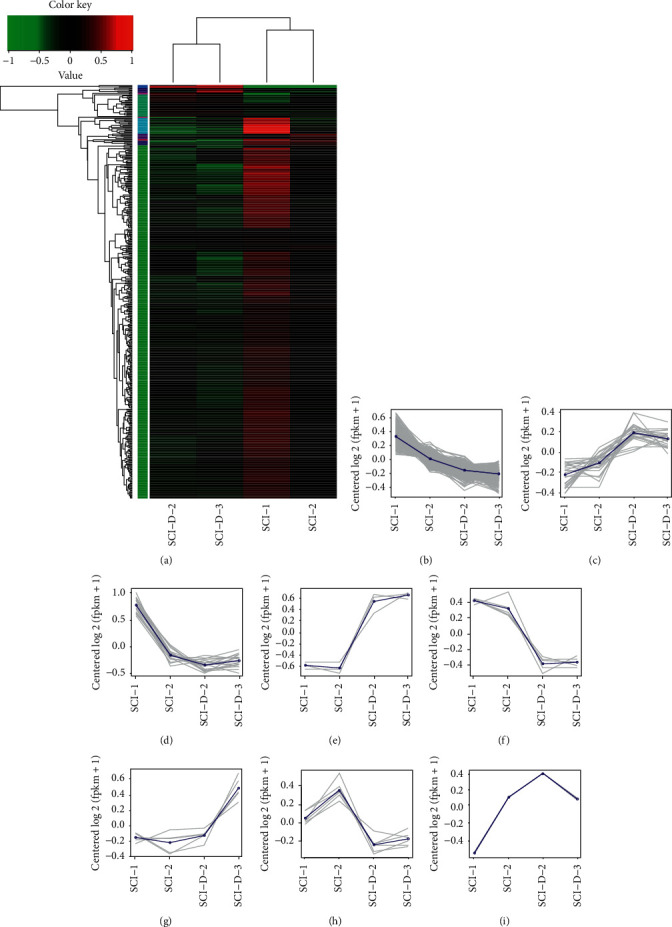
Cluster analysis of DEGs between each spinal cord sample. (a) Hierarchical clustering of DEGs. Red represents high-expression DEGs, and green represents low-expression DEGs. (b)–(i) Subcluster analysis of DEGs. The grey lines show the genes with a similar correlation level, and the blue lines show the mean level.

In the RNA-seq analysis, the expression level of the gene was calculated by aligning the clean reads to the reference genomic region. The FPKM value (fragments per kilobase million, the number of kilobase fragments of 1 gene per million fragments) of each gene in the sample was calculated based on the alignment result and combined with the gene annotation information of the mRNA. The FPKM value was subsequently used as the expression level of the gene in the sample. Nearly 17,195, 17,095, 17,211, and 17,114 expressed genes were identified in SCI-CON1, SCI-CON2, SCI-DRUG1, and SCI-DRUG2 samples, respectively. Then, 443 DEGs (415 upregulated genes and 27 downregulated genes in the experiment group) were screened out. The expression pattern clustering analysis was then performed on DEGs, in which DEGs with the same or similar expression patterns in the same groups were clustered, and the result was visualized in a heat map (Figure 6(a)). All these DEGs were further grouped into 6 subclusters. DEGs from cluster 1 included 1378 SCI high-expression genes (Figure 6(b)), DEGs from cluster 2 included 224 SCI high-expression genes (Figure 6(c)), DEGs from cluster 3 included 317 SCI high-expression genes (Figure 6(d)), DEGs from clusters 4 included 43 SCI high-expression genes (Figure 6(e)), DEGs from cluster 5 included 55 SCI high-expression genes (Figure 6(f)), and DEGs from clusters 6 included 65 SCI high-expression genes (Figure 6(g)).

### 3.5. Functional Annotation and Enrichment Analysis of DEGs

**Figure 7:**
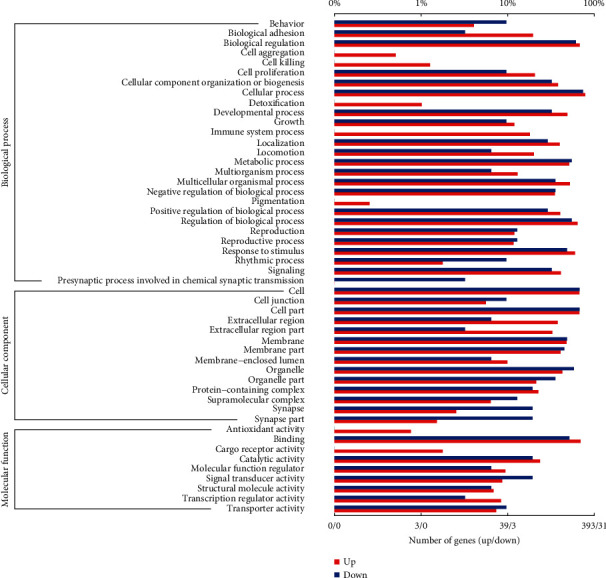
GO terms are classified into 3 categories, namely, biological process, cellular component, and molecular function; the red columns represent the upregulated genes, and the blue columns represent the downregulated genes. The length of the column represents the number of genes annotated to a certain GO term/the proportion of all GO-annotated genes.

**Figure 8:**
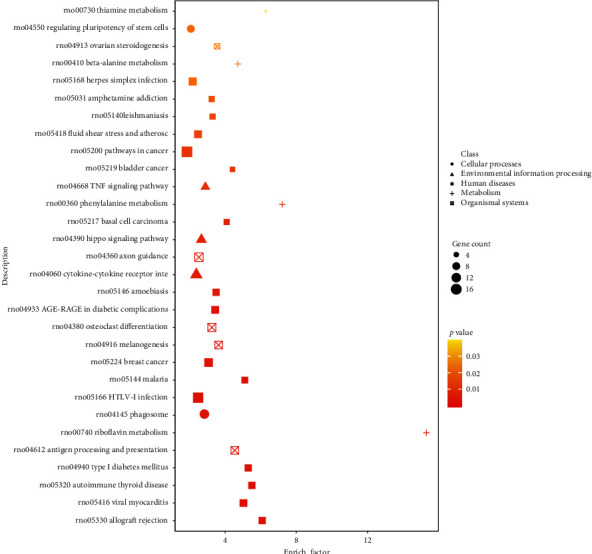
KEGG pathway enrichment analysis of DEGs. The bubble size reflects the gene counts enriched in each term, and color represents the *p* value. Symbols in different shapes represent the cellular processes, environmental information processing, human diseases, metabolism, and organismal systems.

**Figure 9:**
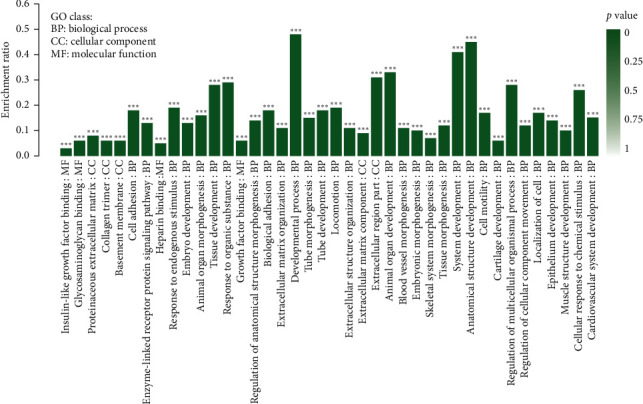
GO enrichment analysis of DEGs. The 40 most significant enriched GO terms were selected as shown in the histogram. Each column represents a GO term; the height of the column indicates the enrichment rate, and darkness of color indicates the significance of the enrichment, which is also reflected as FDR, shown as ^*∗∗∗*^ when FDR <0.001.

The GO database was used to classify differentially expressed genes according to the biological processes they participate in, the components that make up the cells, and their molecular functions. The GO annotation analysis was performed and the result showed that 27 biological processes including locomotion, metabolic process, and the presynaptic process involved in chemical synaptic transmission as well as 14 cellular components including antioxidant activity and 9 molecular functions including the synapse part were annotated (Figure 7). The GO enrichment analysis of the DEGs identified the 30 most enriched GO terms as shown in Figure 8, which included blood vessel morphogenesis, skeletal system morphogenesis, locomotion, developmental processes, and tissue development. In the KEGG enrichment analysis, the most important biochemical metabolic pathways and signal transduction pathways involved in DEGs including thiamine metabolism, Hippo signaling pathway, and axon guidance were identified (Figure 9 and Figure S2).

### 3.6. Differential Expression Analysis of LncRNAs and Construction of the cis/trans-Regulation of LncRNA/mRNA

**Figure 10:**
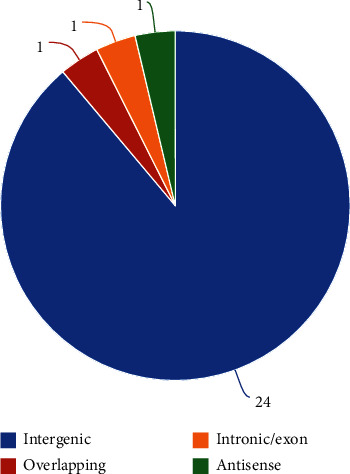
The pie chart shows the proportion of DELs in each kind.

**Figure 11:**
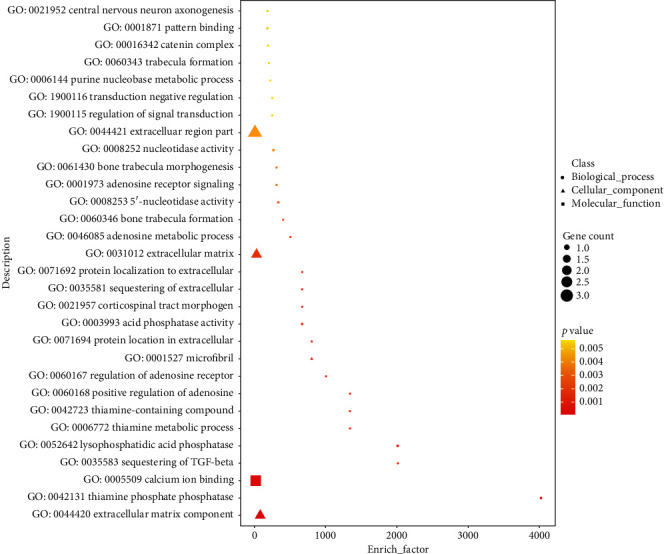
GO enrichment analysis of cis-regulated genes of DELs. The bubble size reflects the gene counts enriched in each term, and darkness of color represents the *p* value. Symbols in different shapes represent the biological process, cellular component, and molecular function.

The expression levels of the differentially expressed LncRNAs were identical to those of the mRNAs; 27 LncRNAs were identified including 24 intergenic, one overlapping, one exon/intronic, and one antisense LncRNA (Figure 10). The predicted target genes were divided into cis-regulation and trans-regulation groups based on the different modes of action of LncRNAs on the target genes. The target genes cis-regulated by LncRNAs were identified in the 10 kb range, upstream and downstream of LncRNA. The expression of genes that transcribed synthetically in the promoter regions was generally promoted, but their expression was inhibited when transcribed in the reversed manner; however, reverse transcription was promoted in some specific cases of the 3′-UTR region. The mRNA of Acpp, Cdh11, Fbn2, Hmcn1, and Prg4 was identified to be cis-regulated by LncRNA AABR07021357.1, AABR07042668.1, AABR07072602.1, AABR07021357.1, and AABR07071383.1, respectively (Table 3). The GO enrichment analysis of the genes that were cis-regulated by the LncRNAs showed that the terms including thiamine phosphate phosphatase, thiamine metabolic compound, central nervous system project, and extracellular matrix regulation were enriched (Figure 11). Trans-regulation was the inhibition of mRNAs by LncRNAs; based on the reverse complementation of LncRNA and mRNA, the target genes were identified by discovering possible hybridization sites of LncRNAs and mRNAs. We found that mRNA Ksr1, Ddi2, Wnt4, Sik1, Acpp, and Nfatc4 were potentially trans-regulated by lncRNA AABR07071383.1 (Table 4).

## 4. Discussion

Troxerutin is a derivative of bioflavonoid rutin that is widely distributed in fruits, vegetables, and grains [17]. There is mounting clinical evidence to show that troxerutin possesses pharmacological effects in the treatment of multiple diseases. It has been revealed that troxerutin improves symptoms of insulin resistance and hyperlipidemia in diabetes via antioxidant activity. Previous studies also showed that troxerutin protects against 2′,2′,4,4′-tetrabromo diphenyl ether (BDE-47) mediated inflammation damage to ameliorate the antioxidant level after traumatic injuries of various pathological conditions such as an acute kidney injury and TBI [11, 18, 19]. Troxerutin has been proven to be beneficial in the treatment of neuropsychological diseases; oral administration of troxerutin could reverse synaptic failure and cognitive disorder in the mice model of Alzheimer's disease [17, 20]. In addition, troxerutin has been shown to be effective in the treatment of hemorrhoidal diseases where it protects the endothelial cells and improves local microcirculation [10, 21]. In a previous study, 15 DEGs were identified by transcriptomic analysis of the blood cells of rats treated with troxerutin [8]; this demonstrated that troxerutin mainly possesses functions of T-cell-mediated cytotoxicity, telencephalon development, cell membrane development, endoplasmic reticulum exports, *β*2-microglobule binding, and transporter associated with antigen presentation (TAP)-binding. Cerebroprotein hydrolysate, also known as Cerebrolysin [22], is suggested to play a role as the precursor to a neurotransmitter; it also directly functions as a neurotransmitter and is used to improve the recovery of neurological function [9, 22, 23]. Due to its ability to penetrate the blood-brain barrier easily, it is widely used in clinical treatment for disorders of the central and peripheral nervous system such as stroke and nerve injuries either separately or in combination with other methods [12, 24]. TCH has been extensively used in clinics in China [10] and has shown to inhibit the process of oxidative stress and promote angiogenesis in cerebral ischemia. It also has an effect on the recovery of TBI by protecting neurovascular units from reoxygenation-induced injury [10].

The goal of the recovery process in SCI is to have a partial or full restoration of motor and sensation nerve conduction [25]; this is very difficult to achieve because there are several factors that accelerate neuron necrosis and impede the functional regeneration of spinal cord neuronal axons [26]. Neurons in the post-SCI microenvironment lack essential growth facilitators [27], such as the signaling pathways involved in axon regeneration, synapse remodeling, and circuit reorganization. Studies have shown that the activation of axon regeneration signaling pathways like WNT and Hippo and the upregulation of attractive and repulsive cues related to molecules in the local environment such as netrins and semaphorins could quicken the recovery sensory deficit and dyskinesia [28–32]. From the results of this study, it can be seen that when the concentration of TCH exceeds 6 ml/kg, the efficacy of TCH decreases and the drug concentration dependence disappears, which indicates that there is a concentration dependence of TCH in a certain concentration range. Therefore, the dose of 6 ml/kg is the balance between the maximum efficacy and the lowest side effects of TCH. In this study, we discovered for the first time that the recommended therapeutic dose of TCH after SCI in rats is 6 ml/kg. Within 2 weeks after administration of this dose of TCH to SCI rats, it was found that further injury could be prevented by reducing neuronal apoptosis and promoting the recovery of neurological function. The GO annotation analysis on the results of RNA-seq suggested that 192 upregulated genes were associated with developmental processes, 162 upregulated genes were associated with signaling, 79 upregulated genes are associated with locomotion, 10 upregulated genes were associated with synapse, and 3 upregulated genes were associated with antioxidant activity. The KEGG enrichment analysis showed that the Rno04390 Hippo signaling pathway and Rno04360 axon guidance were highly enriched. 4 DEGs were considered to be involved in the TCH effect; troxerutin may have downregulated the expression of caspase-6 (Casp6), a downstream enzyme in the activation cascade. The reactivation of Casp6 was found to have a strong correlation to cell death and specific roles in the CNS, especially in the case of neurodegenerative disorders such as axonal degeneration and Alzheimer's disease [33]. It has also been observed that the inhibition or knockdown of Casp6 would delay the course of axon damage and neurofilament dissolution [34]. Bone morphogenetic protein 7 (Bmp7) induces ectopic bone formation by potently inhibiting TNF-*α*-induced oligodendrocyte apoptosis [35]. It is also highly expressed in a number of glial cells and motor neurons after spinal cord injury, which indicates that the upregulation of Bmp7 can promote nerve repair; previous studies have confirmed that local injection of Bmp7 can promote both neuronal regeneration after spinal cord injury and the recovery of motor function in rats [36]. Netrin-1 (Ntn1) has a strong chemotropic function for axonal guidance, cell migration, morphogenesis, and angiogenesis in CNS [32]. In the peripheral nervous system, Ntn1 receptors are expressed in Schwann cells, the cell bodies of sensory neurons, and the axons of both motor and sensory neurons that cause dissociation of UNC5C in microtubules and lead to axon repulsion [31, 32]; Ntn1 also promotes the projection of axon in DRG towards the spinal cord [37]. It could also prevent the initiation of apoptosis; studies show that Ntn1 is downregulated after an axon injury and that the application of exogenous netrin-1 after TBI normalizes spine density and presynaptic excitability of the injured neurons [32]. However, its expression is upregulated after a peripheral nerve transection injury [31].

The other challenge in the recovery of SCI is that the constriction of blood vessels by the action of pericytes leads to acute ischemia [38]. In the long term, pericyte 5-HT1 and *α*2 adrenergic receptors become activated, which would also constrict capillaries, thereby causing a chronic state of ischemia and hypoxia locally for several months as seen in a rat SCI model [39, 40]. It has also been proven that increased blood vascular density and restored blood supply could promote neuronal survival, axonal regeneration, and functional recovery. The results of the present study showed that TCH stabilized the injured segment by reducing posttraumatic spinal cord edema. The GO annotation in RNA-seq results suggested that 129 upregulated genes were associated with the extracellular region part, 138 upregulated genes are associated with negative regulation of biological processes, 79 upregulated genes were associated with locomotion, and 81 upregulated genes were associated with cell proliferation. The GO enrichment analysis showed that GO: 0048514 (blood vessel morphogenesis) and GO: 0009888 (tissue development) were highly enriched. Three DEGs were considered to be involved in the TCH effect, and Cyr61 is a secreted protein that interacted with heparan sulfate proteoglycan and integrins. The expression of Cyr61 was induced by growth factor and its high expression played a role in cell proliferation, angiogenesis, and extracellular matrix formation. Various mechanical stresses induce Cyr61 expression in cartilage/bone tissues and in periodontal ligaments. Cyr61 also engages in a distinct intracellular signaling cascade in microglia/macrophages and promotes M1 macrophage recruitment in the compressed spinal cord [41]. Our present data indicated that Cyr61 was significantly upregulated in the chronically, severely compressed spinal cord and that it colocalized extensively with reactive astrocytes at sites of inflammation. SMOC-2 is highly expressed during wound healing; it could stimulate endothelial cell proliferation and angiogenic activity, thus serving as a target for angiogenesis in myocardial ischemia [42]. Foxc2 plays a role in the development of mesenchymal tissues by acting as a crucial modulator during both angiogenesis and lymphangion genesis. It has been revealed that the expression of Foxc2 was elevated by hypoxia in the postischemia/reperfusion injury in the kidneys; it facilitates vascular development and modulates a variety of angiogenic factors, including Mmp2, Pdgfb, Vegf, Dll4, Notch1, and Hey2 [43]. It can also regulate the expression of osteopontin, which is a determinant of PI-induced tumor angiogenesis. Foxc2 is also found to activate the ERK or PI3K signaling pathway in the bone marrow mesenchymal stem cell-induced angiogenesis [44].

**Figure 12:**
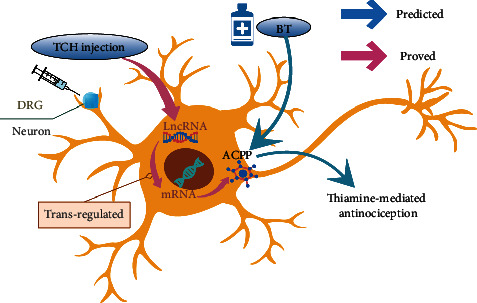
Schematic diagram of the hypothetic molecular mechanism of TCH injection having a synergistic effect to benfotiamine analgesic action after SCI.

In the present study, a total of 21 upregulated and 7 downregulated DELs were identified in the TCH treated group after SCI as compared to the control group. The regulation analysis of LncRNA-mRNA revealed that mRNA of Ksr1, Ddi2, Wnt4, Sik1, Acpp, and Nfatc4 were potentially trans-regulated by LncRNA AABR07071383.1 while Prg4, Cdh11, Fbn2, Hmcn1, and Acpp were cis-regulated by LncRNA AABR07021357.1, AABR07042668.1, AABR07072602.1, AABR07021357.1, and AABR07071383.1, respectively. We focused our attention on the trans/cis-regulation pair of LncRNA AABR07071383.1 and mRNA Acpp. Benfotiamine (S-benzoylthiamine O-monophosphate, BT) was observed to have antinociceptive effects in animals and Acpp (Prostatic acid phosphatase) could dephosphorylate BT in the DRG neurons of the spinal cord. The dephosphorylated product S-benzoylthiamine (S-BT) decomposes to O-benzoylthiamine (O-BT) and finally to thiamine, thus showing antinociceptive effects in animals and humans [45–47]. Our results suggested that TCH may have a synergistic effect with benfotiamine as TCH improves the expression of LncRNA AABR07071383.1, which upregulates the expression of Acpp and accelerates the metabolic process of benfotiamine, thus having antinociceptive effects in the post-SCI condition (Figure 12).

However, a limitation of this study was that the sample size for RNA-seq was small (*n* = 2 for each group); this was due to the requirement of high-quality data for bioinformatic analysis, which was hard to obtain. However, it did not impact the analysis for DEGs and DELs or the function analysis as it has been shown that the false positive rate of transcriptome analysis does not decrease substantially as the sample size increases in the condition of *n* ≥ 2, with the proper depth of sequencing [48], which was qualified in the study.

In conclusion, the findings of this study suggested that TCH injection is effective for the recovery of neural function and local angiogenesis in rats after SCI; the transcriptome analysis showed that TCH regulated the expression of genes that mediate multiple functions such as axon regeneration and blood vessel morphogenesis in the spinal cord tissue. The construction of the LncRNA-mRNA networks showed that TCH mediated the expression of Acpp by upregulating LncRNA, which implies the potential synergistic effect of abirritation that TCH and BT may have for the treatment of SCI; this provides a theoretical basis for the use of TCH injection in clinical practice and investigation.

## Figures and Tables

**Table 1:** Statistics of data before and after quality trimming of raw data.

Sample	Sequences	Bases	Error %	*Q*20%	*Q*30%	GC%
SCI-C-1R	108084164	16212624600	0.0299	95.95	90.08	49.14
SCI-C-1C	107979998	15941090910	0.0294	96.23	90.42	49.00
SCI-C-2R	103441860	15516279000	0.0294	96.18	90.48	48.26
SCI-C-2C	103386392	15294424679	0.0289	96.43	90.78	48.15
SCI-D-1R	106242876	15936431400	0.0298	95.99	90.14	48.30
SCI-D-1C	106183590	15680311454	0.0293	96.26	90.47	48.17
SCI-D-2R	96150788	14422618200	0.0302	95.86	89.82	48.62
SCI-D-2C	96101130	14241154777	0.0298	96.07	90.08	48.52

*Q*20, *Q*30%: the proportion of the number of the bases that have a Phred value higher than 20 and 30, respectively in all bases; Error %: error rate of the bases; and GC%: the proportion of the sum of base G and base C in the total base amount.

**Table 2:** Results of the transcriptome sequencing data aligning to the reference genome.

Sample	All read	Mapped read	Mapped ratio (%)	Unique-read	Unique ratio (%)
SCI1	53989999	47858185	88.64	44192826	81.85
SCI2	51693196	43128530	83.43	40076645	77.53
D1	53091795	46719639	88.00	43390415	81.73
D2	48050565	42540279	88.53	38887230	80.93

All read: the amount of the reads after filtering; Mapped read: the amount of the reads that can be recognized in the reference transcriptome; Mapped ratio: the ratio of the recognized reads in all reads; Unique-read: the amount of the reads that can only be recognized in one unique site in the reference transcriptome; and Unique-ratio: the ratio of the uniquely recognized reads in all reads.

**Table 3:** Analysis of the cis-regulation pairs of DELs-DEGs.

lncRNA	mRNA	Cor	Class	Distance
AABR07021357.1	Prg4	0.999619225	Upstream	−6521
AABR07042668.1	Cdh11	0.99203388	Downstream	75
AABR07072602.1	Fbn2	0.981304859	Downstream	34591
AABR07021357.1	Hmcn1	0.870894572	Downstream	50107
AABR07071383.1	Acpp	0.863110323	Upstream	−3704

Cor: the correlation of each LncRNA-mRNA pair; Class: the classification of the relation between each LncRNA-mRNA pair; and Distance: the distance between each LncRNA-mRNA pair.

**Table 4:** Analysis of the trans-regulation pairs of DELs-DEGs.

lncRNA	mRNA	Cor	Energy
AABR07071383.1	Sik1	0.931430977977652	−93.3
AABR07030791.1	Prss35	0.939241957304881	−90.2
AABR07071383.1	Wnt4	0.955294363494347	−97.4
AABR07071383.1	Ddi2	0.969257491456494	−66.7
AABR07071383.1	Ksr1	0.986526459451311	−88.2
AABR07071383.1	Acpp	0.86311032343114	−83
AABR07071383.1	Nfatc4	0.835789276226398	−87.6

Cor: the correlation of each LncRNA-mRNA pair; Energy: binding energy of each LncRNA-mRNA pair.

## Data Availability

The datasets used and/or analyzed during the current study are available from the corresponding author on reasonable request.

## Abbreviations

SCI: Spinal cord injury
TCH: Troxerutin and cerebroprotein hydrolysate
RNA-seq: RNA sequencing
DEGs: Differentially expressed genes
LncRNA: Long noncoding RNA
DELs: Differentially expressed LncRNAs
KEGG: Kyoto Encyclopedia of Genes and Genomes
GO: Gene Ontology
CNS: Central nervous system
TBI: Traumatic brain injury
BBB scale: Basso, Beattie, and Bresnahan scale
Control/SCI-CONs: SCI control group
Sham: Sham operation control group
WW: Wet weight
DW: Dry weight
Q: Quality value
FPKM value: Fragments per kilobase million
Casp6: Caspase-6
Bmp7: Bone morphogenetic protein 7
Ntn1: Netrin-1.

## Supplementary Materials

Supplementary Materials Figure S1: the bases of the reads are arranged in the 5′ to 3′ direction on the horizontal axis, and the vertical axis is the base quality value of the reads, in which the yellow area represents the range of the quartile value of the mass. The range of the vertical blackline “I” represents the quality of bases in all the reads, and the bold line represents the median of the mass value. Figure S2: directed acyclic graph (DAG) for DEGs of GO enrichment analysis.

## References

[1] Walker M. D. (1991). Acute spinal-cord injury. *New England Journal of Medicine*.

[2] Jain N. B., Harris M. B., Garshick E. (2015). Trends in traumatic spinal cord injury-reply. *JAMA*.

[3] El Masri W. S., Kumar N. (2011). Traumatic spinal cord injuries. *Lancet (London, England)*.

[4] van Middendorp J. J., Hosman A. J., Donders A. R. T. (2011). A clinical prediction rule for ambulation outcomes after traumatic spinal cord injury: a longitudinal cohort study. *The Lancet*.

[5] Ramer L. M., Ramer M. S., Bradbury E. J. (2014). Restoring function after spinal cord injury: towards clinical translation of experimental strategies. *The Lancet Neurology*.

[6] Ajiboye A. B., Willett F. R., Young D. R. (2017). Restoration of reaching and grasping movements through brain-controlled muscle stimulation in a person with tetraplegia: a proof-of-concept demonstration. *The Lancet*.

[7] Stenudd M., Sabelström H., Frisén J. (2015). Role of endogenous neural stem cells in spinal cord injury and repair. *JAMA Neurology*.

[8] Malinska H., Huttl M., Oliyarnyk O. (2019). Beneficial effects of troxerutin on metabolic disorders in non-obese model of metabolic syndrome. *PLoS One*.

[9] An L., Han X., Li H. (2016). Effects and mechanism of cerebroprotein hydrolysate on learning and memory ability in mice. *Genetics and Molecular Research: GMR.*.

[10] Zhao H., Liu Y., Zeng J., Li D., Zhang W., Huang Y. (2018). Troxerutin and cerebroprotein hydrolysate injection protects neurovascular units from oxygen-glucose deprivation and reoxygenation-induced injury in vitro. *Evidence-based Complementary and Alternative Medicine: ECAM*.

[11] Zhào H., Liu Y., Zeng J., Li D., Huang Y. (2018). Troxerutin cerebroprotein hydrolysate injection ameliorates neurovascular injury induced by traumatic brain injury-via endothelial nitric oxide synthase pathway regulation. *International Journal of Neuroscience*.

[12] Ma W., Wang S., Liu X. (2019). Protective effect of troxerutin and cerebroprotein hydrolysate injection on cerebral ischemia through inhibition of oxidative stress and promotion of angiogenesis in rats. *Molecular Medicine Reports*.

[13] Stepanova O. V., Voronova A. D., Chadin A. V. (2019). Efficiency of human olfactory ensheathing cell transplantation into spinal cysts to improve mobility of the hind limbs. *Stem Cells and Development*.

[14] Hurt J. K., Coleman J. L., Fitzpatrick B. J. (2012). Prostatic acid phosphatase is required for the antinociceptive effects of thiamine and benfotiamine. *PLoS One*.

[15] Ge R., Zhu Y., Diao Y., Tao L., Yuan W., Xiong X.-c. (2013). Anti-edema effect of epigallocatechin gallate on spinal cord injury in rats. *Brain Research*.

[16] Park Y.-S., Kim S., Park D.-G. (2019). Comparison of library construction kits for mRNA sequencing in the Illumina platform. *Genes & Genomics*.

[17] Lu J., Wu D. M., Zheng Z. H., Zheng Y. L., Hu B., Zhang Z. F. (2011). Troxerutin protects against high cholesterol-induced cognitive deficits in mice. *Brain : A Journal of Neurology*.

[18] Zhang Z.-F., Zhang Y.-q., Fan S.-H. (2015). Troxerutin protects against 2,2′,4,4′-tetrabromodiphenyl ether (BDE-47)-induced liver inflammation by attenuating oxidative stress-mediated NAD+-depletion. *Journal of Hazardous Materials*.

[19] Shan Q., Zheng G. H., Han X. R. (2018). Troxerutin protects kidney tissue against BDE-47-induced inflammatory damage through CXCR4-TXNIP/NLRP3 signaling. *Oxidative Medicine and Cellular Longevity*.

[20] Babri S., Mohaddes G., Feizi I. (2014). Effect of troxerutin on synaptic plasticity of hippocampal dentate gyrus neurons in a *β*-amyloid model of Alzheimer׳s disease: an electrophysiological study. *European Journal of Pharmacology*.

[21] Werner I., Guo F., Kiessling A. H. (2015). Treatment of endothelial cell with flavonoids modulates transendothelial leukocyte migration. *Phlebology: The Journal of Venous Disease*.

[22] Ziganshina L. E., Abakumova T., Vernay L. (2017). Cerebrolysin for acute ischaemic stroke. *The Cochrane Database of Systematic Reviews*.

[23] Liu Z., Wang W., Huang T. (2019). CH(II), a cerebroprotein hydrolysate, exhibits potential neuro-protective effect on Alzheimer’s disease. *PLoS One*.

[24] Bornstein N. M., Guekht A., Vester J. (2018). Safety and efficacy of Cerebrolysin in early post-stroke recovery: a meta-analysis of nine randomized clinical trials. *Neurological Sciences*.

[25] Rosenzweig E. S., Salegio E. A., Liang J. J. (2019). Chondroitinase improves anatomical and functional outcomes after primate spinal cord injury. *Nature Neuroscience*.

[26] Anderson M. A., O’Shea T. M., Burda J. E. (2018). Required growth facilitators propel axon regeneration across complete spinal cord injury. *Nature*.

[27] Tran A. P., Warren P. M., Silver J. (2018). The biology of regeneration failure and success after spinal cord injury. *Physiological Reviews*.

[28] Gao Y., Bai C., Zheng D. (2016). Combination of melatonin and Wnt-4 promotes neural cell differentiation in bovine amniotic epithelial cells and recovery from spinal cord injury. *Journal of Pineal Research*.

[29] Qu J., Zhao H., Li Q. (2018). MST1 suppression reduces early brain injury by inhibiting the NF-kappaB/MMP-9 pathway after subarachnoid hemorrhage in mice. *Behavioural Neurology*.

[30] Chen J., Laramore C., Shifman M. I. (2017). The expression of chemorepulsive guidance receptors and the regenerative abilities of spinal-projecting neurons after spinal cord injury. *Neuroscience*.

[31] Dun X. P., Parkinson D. B. (2017). Role of netrin-1 signaling in nerve regeneration. *International Journal of Molecular Sciences*.

[32] Nagendran T., Larsen R. S., Bigler R. L. (2017). Distal axotomy enhances retrograde presynaptic excitability onto injured pyramidal neurons via trans-synaptic signaling. *Nature Communications*.

[33] Woo V., Cheng C., Duraikannu A. (2018). Caspase-6 is a dispensable enabler of adult mammalian axonal degeneration. *Neuroscience*.

[34] Chen G., Luo X., Qadri M. Y., Berta T., Ji R.-R. (2018). Sex-dependent glial signaling in pathological pain: distinct roles of spinal microglia and astrocytes. *Neuroscience Bulletin*.

[35] Arriagada R., Lê M. G., Guinebretière J.-M., Dunant A., Rochard F., Tursz T. (2003). Late local recurrences in a randomised trial comparing conservative treatment with total mastectomy in early breast cancer patients. *Annals of Oncology*.

[36] Wang X., Xu J. M., Wang Y. P., Yang L., Li Z. J. (2016). Protective effects of BMP-7 against tumor necrosis factor *α*-induced oligodendrocyte apoptosis. *International Journal of Developmental Neuroscience*.

[37] Sun H., Liu T., Zhu D. (2017). HnRNPM and CD44s expression affects tumor aggressiveness and predicts poor prognosis in breast cancer with axillary lymph node metastases. *Genes, Chromosomes and Cancer*.

[38] Jia Q., Wu W., Wang Y. (2018). Local mutational diversity drives intratumoral immune heterogeneity in non-small cell lung cancer. *Nature Communications*.

[39] D’Amico J. M., Li Y., Bennett D. J., Gorassini M. A. (2013). Reduction of spinal sensory transmission by facilitation of 5-HT1B/D receptors in noninjured and spinal cord-injured humans. *Journal of Neurophysiology*.

[40] Lee J.-S., Fang S.-Y., Roan J.-N., Jou I.-M., Lam C.-F. (2016). Spinal cord injury enhances arterial expression and reactivity of *α*1-adrenergic receptors-mechanistic investigation into autonomic dysreflexia. *The Spine Journal*.

[41] Takano M., Kawabata S., Komaki Y. (2014). Inflammatory cascades mediate synapse elimination in spinal cord compression. *Journal of Neuroinflammation*.

[42] Rocnik E. F., Liu P., Sato K., Walsh K., Vaziri C. (2006). The novel SPARC family member SMOC-2 potentiates angiogenic growth factor activity. *Journal of Biological Chemistry*.

[43] Wang T., Zheng L., Wang Q., Hu Y.-W. (2018). Emerging roles and mechanisms of FOXC2 in cancer. *Clinica Chimica Acta*.

[44] Ogura Y., Miyake N., Kou I. (2015). Identification of HOXD4 mutations in spinal extradural arachnoid cyst. *PLoS One*.

[45] Nacitarhan C., Minareci E., Sadan G. (2014). The effect of benfotiamine on mu-opioid receptor mediated antinociception in experimental diabetes. *Experimental and Clinical Endocrinology & Diabetes*.

[46] Ang C. D., Alviar M. J., Dans A. L. (2008). Vitamin B for treating peripheral neuropathy. *The Cochrane Database of Systematic Reviews*.

[47] Miranda-Massari J. R., Gonzalez M. J., Jimenez F. J., Allende-Vigo M. Z., Duconge J. (2011). Metabolic correction in the management of diabetic peripheral neuropathy: improving clinical results beyond symptom control. *Current Clinical Pharmacology*.

[48] Robles J. A., Qureshi S. E., Stephen S. J., Wilson S. R., Burden C. J., Taylor J. M. (2012). Efficient experimental design and analysis strategies for the detection of differential expression using RNA-sequencing. *BMC Genomics*.

