# Supplementary material for: Transcriptome Analysis Reveals the Effects of Troxerutin and Cerebroprotein Hydrolysate Injection on Injured Spinal Cords in Rats
Source: Evid Based Complement Alternat Med. 2020 Aug 4;2020:3561235. doi: 10.1155/2020/3561235 (PMC7424371; doi:10.1155/2020/3561235)
Supplement: Supplementary Materials — Figure S1: the bases of the reads are arranged in the 5′ to 3′ direction on the horizontal axis, and the vertical axis is the base quality value of the reads, in which the yellow area represents the range of the quartile value of the mass. The range of the vertical blackline “I” represents the quality of bases in all the reads, and the bold line represents the median of the mass value. Figure S2: directed acyclic graph (DAG) for DEGs of GO enrichment analysis. [file 3561235.f1.pdf]

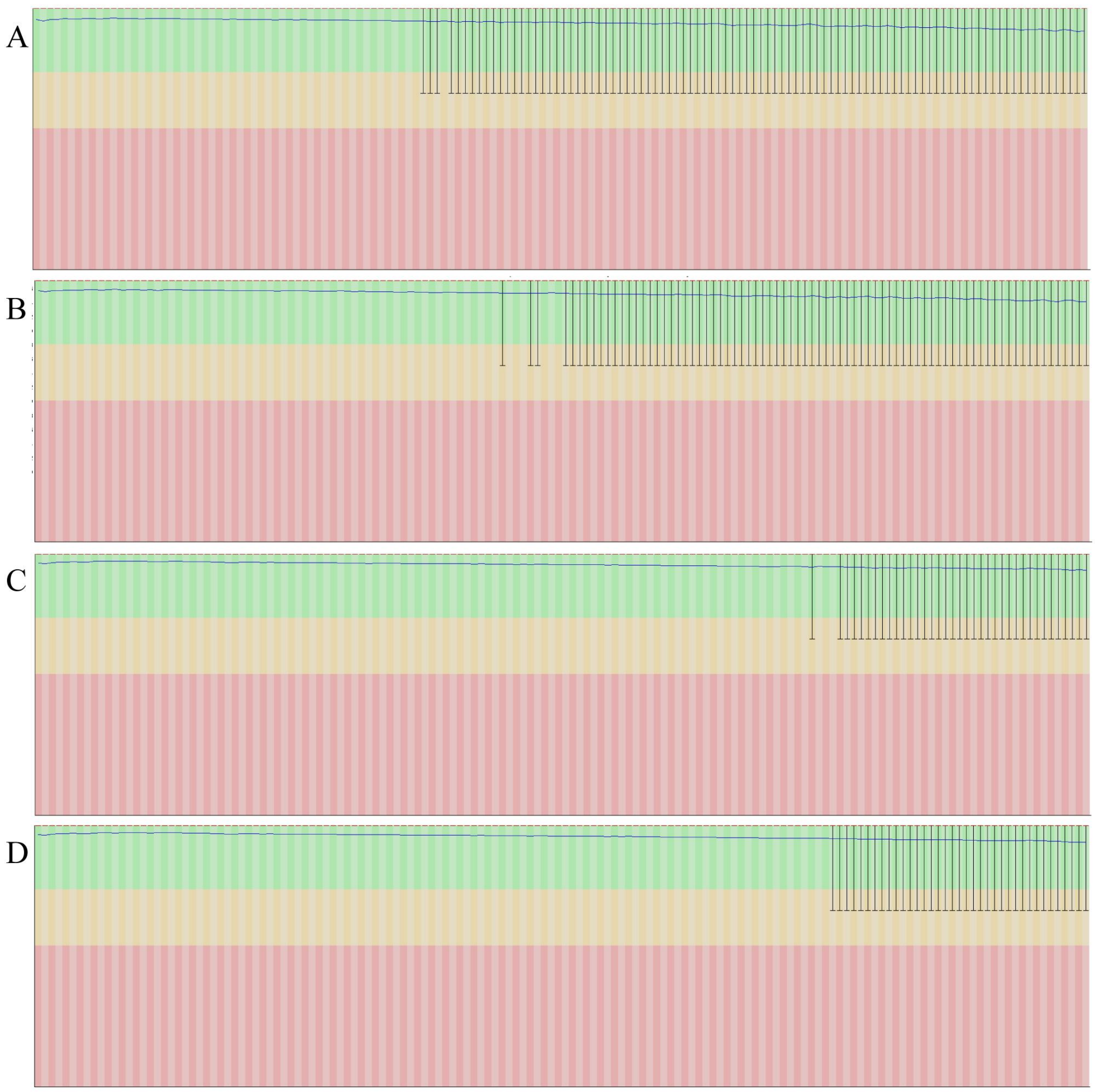

Figure S1.

The bases of the reads are arranged in the 5' to 3' direction on the horizontal axis, and the vertical axis is the base quality value of the reads, in which the yellow area represents the range of the quartile value of the mass. The range of the vertical black line "I" represents the quality of bases in all the reads and the bold line represents the median of the mass value.

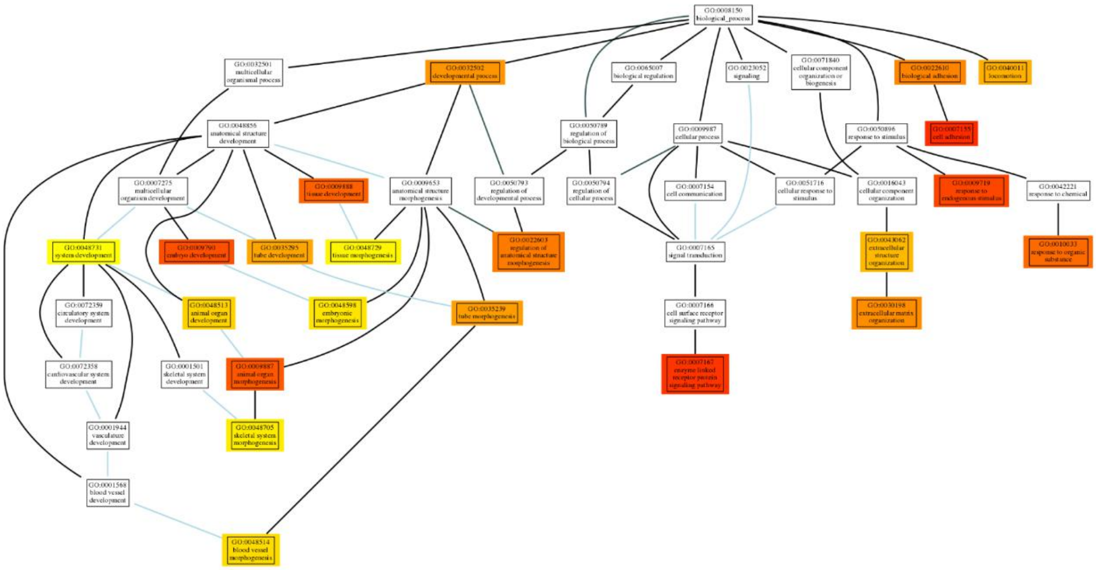

Figure S2.

Directed acyclic graph (DAG) for DEGs of GO enrichment analysis.
